# Are Helicobacter Pylori and Other Helicobacter Species Infection Associated with Human Biliary Lithiasis? A Meta-Analysis

**DOI:** 10.1371/journal.pone.0027390

**Published:** 2011-11-08

**Authors:** Di Zhou, Yong Zhang, Wei Gong, Sayid Omar Mohamed, Henry Ogbomo, Xuefeng Wang, Yingbin Liu, Zhiwei Quan

**Affiliations:** 1 Department of General Surgery, School of Medicine, Xinhua Hospital, Shanghai JiaoTong University, Shanghai, People's Republic of China; 2 Departments of Oncology, Biochemistry and Molecular Biology, Faculty of Medicine, Southern Alberta Cancer Research Institute, University of Calgary, Calgary, Alberta, Canada; Indian Institute of Science, India

## Abstract

**Background:**

Since the isolation of *Helicobacter* species in biliary system, a hypothetical question was raised about the role of these agents in the development of cholelithiasis. This meta-analysis is to explore the association between the *Helicobacter* infection and biliary lithiasis.

**Methodology/Principal Findings:**

A systematic literature search was performed to identify all eligible articles. Meta-analysis which was carried out using odds ratio and random effect model, 95% confidence intervals for odds ratio was calculated. Quantitative assessment of heterogeneity was explored by chi-square test with significance set at P value 0.10 and was measured using *I* ^2^ statistic. Eighteen studies published between 1998 and 2011 were finally eligible for meta-analysis. *H. Pylori, H. Bilis, H. Hepaticus, H. Pullorum* and *H. Ganmani* were studied. With heterogeneity (*I^2^* = 69.5%, P<0.0001), significantly higher pooled infection rates of *H. Pylori* (OR: 2.59, 35.82% versus 26.75%, P = 0.01) and *H. Hepaticus* (OR: 3.13, 31.30% versus 12.12%, P = 0.02) were observed in lithiasis group. Higher prevalence of *H. Pylori* in cholelithiasis patients were reported by studies from East Asia, South Asia and South America. Evidences supporting the higher presence of *H. Pylori* in cholelithiasis patients could be found by PCR for detecting 16s rRNA in bile, 26kDa protein gene in biliary tissue and immunohistochemistry. Using multiple detection tests could increase the detection rate of *H. Pylori*.

**Conclusions/Significances:**

Our meta-analysis suggests a trend of higher presence of *H. Pylori* in cholelithiasis patients than control group and this trend was significant in the regions with higher prevalence of this agent. Evidences supporting the association between *Helicobacter* and cholelithiasis could be found by using different tests but the gold standard for the identification of these bacteria in biliary system has yet to be established. Considering obvious heterogeneity, a large multi-center study will facilitate us to further clarify the association between the *Helicobacter* infection and cholelithiasis.

## Introduction

*Helicobacter Pylori* (*H. Pylori*), classified as a type I carcinogen, is proved to be the main pathogen responsible for chronic gastritis, gastroduodenal ulcers and gastric carcinoma [1]. Besides this most well-known member, more than 20 other *Helicobacter* species (*Helicobacter* sp.) have been identified and several of them are associated with various chronic digestive diseases [2]–[4]. Biliary lithiasis is one of the most common conditions requiring surgical management. Although its pathogenesis still remains obscure, chronic infection is already accepted as a potential risk factor [5]. In the past decade, with continuous isolation of *H. Pylori*, *H. Bilis*, *H. Hepaticus* and other *Helicobacter* sp. in bile, biliary tract tissue and stone specimens [6], a hypothetical question was raised about the role of these infectious agents in the development of biliary lithiasis.

However, studies carried out so far failed to establish association between the colonization of *Helicobacter* sp. in biliary tract and stone diseases. Some reported strong positive correlation while others indicated totally negative results [7]–[9]. To understand these controversial findings, the following three premises should be concerned. Firstly, most studies on this issue were observational reports without control groups. When it comes to case-control studies, many of them have involved either small sample size of lithiasis patients, or even smaller members of controls [10], [11]. Insufficient sample size may not have enough power to distinguish the differences between the two groups. Secondly, discordant results may be due to the geographic distribution variations of *Helicobacter* species. Epidemiologically, The prevalence of *H. Pylori* in East Asian countries is significantly higher than in western countries [12]. Investigators from Taiwan obtained DNA fragments of *Helicobacter* sp. in patients with extrahepatic biliary disorders by polymerase chain reaction (PCR) while authors from German failed to achieve comparable results using the same detecting method [13]–[15]. Moreover, various techniques are utilized for identify *Helicobacter* species. Among them, PCR is the most common used means for detecting 16s rRNA, the genes encoding the *H. Pylori-*specific 26kDa protein, urease A, Vac A and Cag A proteins. Besides PCR, many data available also have relied on culture, immunohistochemistry staining (IHC), fast urease test (FUT) and enzyme-linked immunosorbent assay (ELISA) [16], [17]. However, the detection rates of *Helicobacter* sp. vary between the above methods and they have not been comprehensively measured or tracked yet.

Based on the current status of knowledge in this field, we systematically review the published studies and present a meta-analysis to explore whether there is an association between the *Helicobacter* sp. infection and biliary lithiasis formation.

## Materials and Methods

### Literature Search

We followed QUOROM guidelines [18] for conducting meta-analysis and the study design and report adhered to the PRISMA Statement guidelines (supporting information Table S1). Two investigators (DZ and YZ) performed a systematic literature search independently by using Pubmed, Embase, ISI databases and the Cochrane Library Central between January 1980 and June 2011 at different time and at two different medical science information centers respectively affiliated to FuDan University and Shanghai JiaoTong University. The search was limited to humans. The search strategy was based on the following Medical Subject Heading terms (MeSH) and text words: "hepatobiliary", "biliary", "biliary tract", "gallbladder", "biliary duct", "lithiasis", "stone", "stone disease", "calculi", "gallstones", "cholelithiasis", "choledocholithiasis", "cholecystolithiasis", "gallbladder stone", "common bile duct stone", "*helicobacter*", "*Helicobacter Pylori*", "*Helicobacter Bilis*", "*Helicobacter Hepaticus*", "*Helicobacter Pullorum*", "*Hp*", "*H. Pylori*", "*H. Bilis*", "*H. Hepaticus*", "*H. Pullorum*", "*H. Ganmani*", "*Helicobacter* species", "*Helicobacter* sp. ", "*Helicobacter* genus" and "case-control". The related articles function and reference lists were used to broaden the search. The investigators and experts in this field ensured that all potentially relevant reports were identified. The search was limited to humans. No restriction was set for languages or date of publication. When further information was required, the corresponding authors of relevant papers were contacted by the reviewers.

### Data Extraction

The above two investigators performed the data extraction independently and in the case of discrepancy, the decision was made by discussion or in consultation with a third author (XFW). A data extraction was carried out to record details of first and correspondent authors, year and country of publication, study type, method of detection, diagnosis, type of specimen, sample size and type of organism identified. The numbers of *Helicobacter*-positive and negative patients in lithiasis group and control group were collected. The number of the relevant patients was extracted for the subgroup analysis stratifying by *Helicobacter* species, geographic regions, types of detecting methods and types of specimens.

### Inclusion Criteria

1. Study design: published case-control study provide raw data dealing with *Helicobacter*-sp. infection in both human lithiasis group and control group.
2. *Helicobacter*-sp. infection had to be confirmed by polymerase chain reaction (PCR), culture, immunohistochemistry staining (IHC), fast urease test (FUT) and enzyme-linked immunosorbent assay (ELISA). At least one positive test was regarded as confirmation of infection.

### Exclusion Criteria

1. Case report and observational studies without control groups.
2. Studies in which the raw data of *Helicobacter*-sp. infection rates were not available in either lithiasis cases group or control group.
3. Studies limited to animals.

### Statistical Analysis

Calculation for dichotomous variables was carried out using the odds ratio (OR) and their 95% CI as the summary statistic. The Mentel-Haenszel method was used to combine OR for the outcome parameters. Yate's correction was performed for studies containing a “zero” in one cell for the number of positive cases in one of the two groups [19].

Owing to the between-study variability of sample size and detection methods, overall estimates were calculated by using the random effect models [20]. Quantitative assessment of heterogeneity was explored by chi-square test with significance set at *P* value 0.10 and was measured using *I* ^2^ statistic. The larger is the value, the greater is the heterogeneity. Graphical test with Begg's funnel plot was used to detect the publication bias [21]. A two-sided *P* value less than 0.05 was considered statistically significant.

The software SAS 9.13 (SAS Institute, Gary, North Carolina), Review Manager version 4.2 (Cochrane Collaboration, Software Update, Oxford), Intercooled Stata version 7.0 for Windows (Stata Corporation, USA) were used for conducting this meta-analysis.

## Results

### Description of Studies

Both of the two investigators agreed on the result of data extraction. The strategy of study selection is displayed in Figure S1. Eighteen case-control studies published between 1998 and 2011 were finally eligible for meta-analysis (Table 1) [8], [9], [22]–[37]. These studies involved 1678 patients with a total *Helicobacter* sp. infection rate of 30.39% (510/1678). The cumulative sample size of biliary lithiasis group was 1071 of which 364 were positive (33.39%) while of 607 controls only 146 (24.05%) were positive for *Helicobacter* infection.

**Table 1:** Characteristics of studies on Helicobacter sp. in cholelithiasis patients and control group.

Reference (year)	Country	Method of Detection	Disease	Specimen	Organism identified	*Helicobacter sp.* (+) in lithiasis Group n/N	*Helicobacte sp.* (+) in Control Group n/N
Figura (1998)	Italy	ELISA (*H.Pylori* IgG)	Cholecystolithiasis	Serum	*H.Pylori*	92/112	90/112
Myung (2000)	Korea	ELISA (*H.Pylori* IgG) PCR (Urea A, 26KD protein) IHC	Hepatolithiasis, Choledocholithiasis	Serum, Bile, Biliary Tissue, Stone	*H.Pylori*	7/30	0/8
Leong (2001)	China	PCR (16sRNA)	Choledocholithiasis	Bile	*Helicobacter sp.*	4/25	0/4
Löhr (2002)	Yugoslavia	PCR (16sRNA)	Cholecystolithiasis	Bile	*H.Pylori*	37/63	3/11
Bulajic (2002)	Yugoslavia	PCR (Urea A)	Cholelithiasis	Bile	*H.Pylori*	26/48	1/7
Matsukura (2002)	Japan	PCR (16sRNA)	Cholecystolithiasis	Bile	*H.bilis*	18/42	4/14
Presser Silvar (2003)	Brazil	PCR (16sRNA) Culture	Cholelithiasis	Bile, Biliary Tissue	*H.Pylori*	28/51	2/18
Chen (2003)	New Zealand	PCR (16sRNA, 26KD protein) ELISA (*H.Pylori* IgG)	Cholecystolithiasis	GB Tissue, Bile, Serum	*H.Pylori*	35/70	15/37
Farshad (2004)	Iran	PCR (16sRNA)	Cholecystolithiasis	Stone, Bile	*H.Pylori*	10/33	0/40
Abayli (2005)	Turkey	PCR (16sRNA) IHC Culture	Cholecystolithiasis	Stone, GB Tissue	*H.Pylori*	18/77	0/20
Kobayashi (2005)	Japan	PCR (16sRNA, Urea A) Culture	Cholelithiasis	Bile	*H.Pylori, H.bilis*	15/30 1/30	2/21 0/21
Bohr (2007)	Germany	PCR (16sRNA) IHC Culture	Cholecystolithiasis	GB Tissue	*H. ganmani*	1/57	0/22
Hamada (2009)	Japan	PCR (16sRNA) Culture	Cholelithiasis	Bile	*H.hepaticus*	25/60	4/32
Griniatsos (2009)	Greece	PCR (16sRNA)	Cholecystolithiasis	GB Tissue	*H.Pylori*	4/89	2/42
Yucebilgil (2009)	Turkey	PCR (16sRNA) Culture	Cholelithiasis	GB Tissue	*H.Pylori*	2/41	13/27
Karagin (2010)	Sweden	PCR (16sRNA)	Cholecystolithiasis	GB Tissue	*H.Pylori, H. pullorum*	1/100 6/100	0/102 0/102
Shimoyama (2010)	Japan	ELISA (*H.hepaticus* IgG)	Cholelithiasis	Serum	*H.hepaticus*	11/55	4/34
Yakoob (2011)	Pakistan	PCR (16sRNA) IHC	Cholelithiasis	Biliary Tissue	*H.Pylori*	23/88	6/56

ELISA: enzyme-linked immunosorbent assay; PCR: polymerase chain reaction; RNA: ribonucleic acid; IHC: immunohistochemistry; Urea A: Urease A; GB: gallbladder.

In the 18 included studies, six were from East-Asian countries, one from South Asia (Pakistan), three from Middle East, one from Oceania (New Zealand), six from Europe and one from South America (Brazil). Totally 5 species of *Helicobacter* including *H. Pylori*, *H. Bilis*, *H. Hepaticus*, *H. Pullorum* and *H. Ganmani* were studied [9], [26], [31], [32], [35], [36]. *H. Pylori* was still the commonest species which was identified in 13 studies (Table 2). As for detecting methods, fourteen studies used PCR for 16s rRNA while two and three studies used that for *H. Pylori*-specific 26kDa protein and urease A. Besides PCR, culture, immunohistochemistry and ELISA were performed in six, three and four studies, respectively.

**Table 2:** Meta-analysis on the prevalence of *Helicobacter species* in cholelithiasis group compared with control group.

Subgroup	No. of Studies	*Helicobacter sp.* (+) in lithiasis Group n/N	*Helicobacter sp.* (+) in Control Group n/N	OR (95% CI)	*p* value
Helicobacter Species					
*H. Pylori*	13	298/832	134/501	2.59 (1.21, 5.55)	0.01*
*H. Bilis*	2	19/72	4/35	1.92 (0.57, 6.46)	0.29
*H.Hepaticus*	2	36/115	8/66	3.13 (1.20, 8.19)	0.02*
*H.Ganmani*	1	1/57	0/22	1.19 (0.05, 30.43)	0.91
*H.Pullorum*	1	6/100	0/102	14.10 (0.78, 253.71)	0.07

OR, odds ratio; CI, confidence interval;

Δ: *p*<0.01;

*: *p*<0.05.

All of the 18 included studies were approved by the Ethics Committee of their respective institute and informed consents were obtained from all patients before their enrolling in the studies. No publications for the assessment of social or ethical issues could be found.

### Subgroup analysis of prevalence of *H. Pylori* in biliary lithiasis group and control group

**Figure 1:**
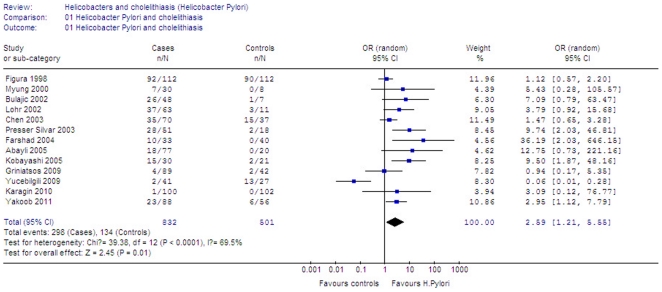
Meta-analysis of studies on the prevalence of *H.Pylori* in cholelithiasis group compared with control group.

Thirteen studies were focused on *H. Pylori* [8], [22], [24], [25], [27]–[31], [33]–[35], [37]. Regardless of various detecting methods, a significantly higher infection rate was noted in lithiasis group than control group (35.82% versus 26.75%, Z = 2.45, P = 0.01). With heterogeneity (*I* ^2^ = 69.5%, P<0.0001), the cumulative odds ratio for the sample was 2.59 (95% CI 1.21–5.55) and favored the role of *H. Pylori* in lithiasis cases (Figure 1). A sensitivity analysis omitting 1 study at a time and calculating the pooled ORs for the remainder of the studies showed that the two study by Yucebilgili [34] and Figura [22] might substantially influence the pooled OR. After excluding these two studies, there was no heterogeneity detected. Subgroup analysis was performed to investigate the influential factors that may impact the overall results. The potential differences in various detecting methods, types of specimens and geographic distribution are the most important concerns.

Four techniques including ELISA [8], [22], [28], PCR [8], [24], [25], [27]–[31], [33]–[35], [37], immunohistochemistry staining (IHC) [30], [37] and culture [27], [30], [31], [34] were utilized for identifying the presence of *H. Pylori* (Table 3). ELISA was performed in three studies in order to detect the *H. Pylori*-specific IgG in serum samples. The pooled data showed that the positive rate of this antibody was similar between the lithiasis group and control group (67.38% versus 69.33%, OR: 1.01, 95% CI: 0.58–1.75, P = 0.97). Because PCR is the most widely used method and different primers may vary in sensitivity and specificity, we analyzed the results based on 16s rRNA, 26kDa protein and urease A genes (Table 3). Two studies by Farshad [29] and Abayli [30] reported that 16s rRNA was found in biliary stones. In bile specimens, the pooled OR of 16s rRNA was 6.74 (95% CI: 2.55–17.83), indicating a significantly higher detection rate in cholelithiasis patients than control group (42.33% versus 7.04%, P = 0.000). When using the primer of urease A, subgroup analysis also revealed a higher presence of 30.54% (51/167) in lithiasis group than that of 16.67% (6/36) in control group, but the difference was not statistically significant (OR: 1.63, 95% CI: 0.64–4.15, P = 0.31). Besides bile, detection was also performed in gallbladder and biliary tract epithelium (Table 3). The ORs of 16s rRNA and 26kDa protein were respectively, 1.39 (95% CI: 0.90–2.16, P = 0.14) and 2.48 (95% CI: 1.05–5.84, P = 0.04), indicating that more 26kDa protein was detected in lithiasis group. Notably, immunohistochemistry staining (IHC) supported the higher presence of *H. Pylori* in lithiasis group than controls (15.17% versus 5.95%, OR: 3.79, 95% CI: 1.43–10.04, P = 0.007). In addition, five studies utilized bile, gallbladder and biliary duct tissues for culture [27], [28], [30], [31], [34]. Unfortunately, from bile specimens, no grown spiral shaped Gram-negative bacteria could be observed by microscopy and only 3.02% (6/199) of the tissue samples from biliary lithiasis were positive for *H. Pylori*. Although the above subgroup analysis revealed great differences, the pooled result of various detection methods showed that the prevalence of *H. Pylori* was significantly higher in lithiaisis group than control group did not change.

**Table 3:** Subgroup analysis on the prevalence of *H.Pylori* in cholelithiasis group compared with control group.

Subgroup	No. of Studies	*H. Pylori* (+) in lithiasis Group n/N	*H. Pylori* (+) in Control Group n/N	OR (95% CI)	*p* value
Geographic Distribution					
East Asia	2	22/60	2/29	8.35 (2.01, 34.69)	0.003Δ
South Asia	1	23/88	6/56	2.95 (1.12, 7.79)	0.03*
Middle East	3	30/151	13/87	2.61 (0.03, 272.54)	0.69
Oceania	1	35/70	15/37	1.47 (0.65, 3.28)	0.35
Europe	5	160/412	96/274	1.72 (0.86, 3.43)	0.12
South America	1	28/51	2/18	9.74 (2.03, 46.81)	0.004Δ
Detecting Methods and Specimens					
Serum					
ELISA (*H. Pylori*-Ig G)	3	126/187	104/150	1.01 (0.58, 1.75)	0.97
Bile					
PCR (16s rRNA)	4	69/163	5/71	6.74 (2.55, 17.83)	0.000Δ
PCR (Urease A)	3	51/167	6/36	1.63 (0.64, 4.15)	0.31
Culture	2	0/67	0/20	N/A	N/A
Biliary Tissue					
PCR (16s rRNA)	8	93/562	38/310	1.39 (0.90, 2.16)	0.14
PCR (26kDa protein)	2	26/123	8/67	2.48 (1.05, 5.84)	0.04*
Culture	4	6/199	0/86	3.73 (0.20, 68.97)	0.38
IHC	3	32/211	5/84	3.79 (1.43, 10.04)	0.007Δ
The Number of *H. Pylori* (+) Tests (including the study by Figura)					
Only 1 test was *H. Pylori* (+)	8	200/537	111/359	2.12 (0.63, 7.15)	0.22
At least 2 tests were *H. Pylori* (+)	5	98/295	23/142	3.18 (1.44, 7.00)	0.004Δ
The Number of *H. Pylori* (+) Tests (excluding the study by Figura)					
Only 1 test was *H. Pylori* (+)	7	108/425	21/247	2.54 (0.50, 12.88)	0.26
At least 2 tests were *H. Pylori* (+)	5	98/295	23/142	3.18 (1.44, 7.00)	0.004Δ

ELISA: enzyme-linked immunosorbent assay; PCR: polymerase chain reaction; RNA: ribonucleic acid; Urea A: Urease A; IHC: immunohistochemistry; OR, odds ratio; CI, confidence interval;

Δ: *p*<0.01;

*: *p*<0.05.

Among the 13 included studies focused on *H. Pylori*, six studies used only one diagnostic test (five used PCR and one used ELISA) while the remaining 7 performed two or more different tests (Table 1). The total detection rate of *H. Pylori* might not only depend on the detection technology itself, but also affected by how many tests were utilized in each study. Therefore, we conducted the subgroup analysis stratified by studies in which only one test was *H. Pylori*-positive and at least two tests were *H. Pylori*-positive. Finally, as showed in Table 3, there were 5 studies in which at least two different tests were positive for *H. Pylori* and subgroup analysis of them indicated a significantly higher total detection rate of *H. Pylori* (33.22% versus 16.20%, P = 0.004) in the lithiasis group than control group (without double counting the same patients who were positive in different tests). Conversely, analysis of studies in which only one test was *H. Pylori*-positive showed comparable detection rates between the two groups (37.24% versus 30.92%, P = 0.22). However, according to these data, the detection rate of studies in which only one test was *H. Pylori*-positive (37.24% in lithiasis group and 30.92% in control group, respectively) was relatively higher than that of studies in which at least two tests were positive (only 33.22% in lithiasis group and 16.20% in control group, respectively). The possible explanation for this result might be due to the type of diagnostic technology and specimen. In the study by Figura [22], ELISA was performed to detect the *H. Pylori*-IgG in serum samples and the detection rates in the lithiasis and control group were 82.14% (92/112) and 80.36% (90/112), respectively. Considering that only detecting IgG in serum might not reflect the real infectious state of *H. Pylori* in biliary system, we conducted further subgroup analysis after excluding this study. Finally, in studies which only one test was *H. Pylori*-positive, the overall result did not change (P = 0.26) but the detection rates were decreased to 25.41% (108/425) in lithiasis group and 8.50% (21/247) in control group, respectively (Table 3).

Stratifying by geographic region, the ORs were 8.35 (95% CI 2.01–34.69) for 2 studies conducted in East Asia [8], [31], 2.95 (95% CI 1.12–7.79) for 1 study from Pakistan [37] and 9.74 (95% CI, 2.03–46.81) for 1 study from Brazil [27]. Authors from these three regions were able to confirm significantly higher infection rates of *H. Pylori* in lithiasis group than control group (P = 0.003, 0.03 and 0.004, respectively). In contrast, investigators from Middle East [29], [30], [34], New Zealand [28] and Europe [22], [24], [25], [33], [35] could not find any difference of *H. Pylori* presence between the two groups (P = 0.69, 0.35 and 0.12, respectively) (Table 3).

### Prevalence of other *Helicobacter* sp. in biliary lithiasis group and control group

Besides *H. Pylori*, the pooled prevalence of *H. Hepaticus* in 2 studies was also much higher in lithiasis group (31.30% versus 12.12%, P = 0.02) [32], [36]. Regarding *H. Bilis*, conversely, analysis of 2 studies did not show any difference between the two groups (26.39% versus 11.43%, P = 0.29) [26], [31]. In addition, there was one study concerning *H. Ganmani* and another one on *H. Pullorum,* either demonstrated similar positive rates between the lithiasis and controls (P = 0.91 and 0.07, respectively) [9], [35]. Unfortunately, we could not conduct further analyses stratified by geographic distribution, detecting methods or types of specimens because only one or two studies reported results of each of these 4 species.

### Publication Bias

**Figure 2:**
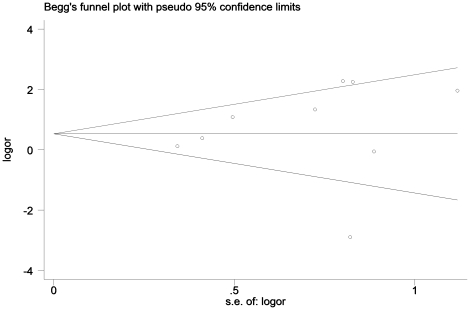
Funnel plot to detect publication bias. s.e. represents standard error.

The funnel plot did not show evidence of publication bias (Begg's test z = 0.73, P = 0.466, continuity corrected). (Figure 2).

## Discussion

The relationship between the *Helicobacter* infection and cholelithiaisis still remains controversial. Some studies have supported a cause-and-effect association while some others even failed to confirm the existence of these bacteria in biliary specimens [7]–[10]. Also, there is a lack of strong evidences to determine the possible entry routes of *Helicobacter* sp. to the hepatobiliary tree including either the ascending duodenum infection or the portal system circulation pathway [38], [39]. Till now, we do not know exactly which *Helicobacter* sp. are to be found in human biliary lithiasis disease and what's the best way to identify their colonization.

Among various *Helicobacter* species, *H. Pylori* still remains the most studied one whose prevalence ranges from 60 to 80% in patients with gastric ulcer and 90–100% in those with duodenal ulcer [1], [40]. In this meta-analysis, a significantly higher presence of *H. Pylori* was observed in lithiasis group than control group (35.82% versus 26.75%, P = 0.01), implicating a potential association between this bacterial infection and lithiasis. However, when regarding each included study, although most of them reported a higher prevalence of *H. Pylori* in lithiasis group, only five reached the significant level [27], [29], [31], [32], [37]. The small sample size might be one of the explanations for the negative results.

At present, PCR, culture, immunohistochemistry and ELISA are the most widely used techniques for detecting *Helicobacter* sp. In our meta-analysis, a wide rage of detection rates vary from 3.02% to 80% was observed owing to different methods and types of specimens. ELISA is an inexpensive and easy test on serum samples to determine the infectious state of *H. Pylori*. However, because of the limitation to the specificity in confirming species and subspecies due to cross-reactivity of the *Helicobacter* and *Campylobacter*, no strong correlation could be established yet between the presence of *Helicobacter* specific-IgG in serum and the presence of *H. Pylori* in biliary system [28], [41]. PCR is a highly sensitive method which can selectively amplify the copies of a target gene by more than 10^6^-fold [42], [43]. Recently, assays based on PCR have been developed to detect the presence of *H. Pylori* DNA by using several gene targets directly from the biopsies. The targets include the 16s rRNA gene, urease A gene and the 26kDa species-specific protein gene [44], [45]. In this study, subgroup analysis was carefully conducted and we found that diverse sensitivities and specificities of these primers as well as the impact of specimen type really contributed to the variation of the pooled estimates (Table 3). The three primers all reported relatively higher positive rates in lithiasis group than control group, however, in bile, it was 16s rRNA reached significant level and in biliary tissue 26kDa favored the role of *H. Pylori* in lithiasis formation. (P = 0.000 and 0.04, respectively). To explain the greatly different results of 16s rRNA between bile and tissue samples, the existed finding suggested that *Helicobacter* species relatively less colonize in biliary epithelium [46]. Among the three primers, urease A may cross-react with the urease gene of other organisms so that PCR based on this primer may produce a false positive result [47]. Therefore, many studies have utilized 16s rRNA and 26kDa gene, which can respectively identify the entire *Helicobacter* genus and the *H. Pylori*-specific subunit of urease rather than being interfered by other species that can produce urease [48]. Till now, only a few studies have confirmed the existence of *H. Pylori* in biliary system by immunohistochemistry. The routine hematoxylin-eosin stain is not well suited for *H. pylori* detection because of the weak contrast between the bacteria and the mucus. The Warthin-Starry stain provides a better visualization of the bacteria, but the procedure is difficult to carry out. This technique is time-consuming and requires instant preparation of the relevant reagents [49]. Notably, meta-analysis of three studies using Warthin-Starry stain demonstrated a higher presence of *H. Pylori* in lithiasis group (P = 0.007). Culture remains the definitive method to prove the viability of *H. Pylori*. Unfortunately, our study revealed that no grown *H. Pylori* from bile could be observed and only 3.02% (6/199) of the tissue samples were positive in cultivation of this bacterium. Why this occurs might be due to the use of frozen bile as sample and the strongly inhibitory effects of bile acid. Obviously, further research on exploring the optimal conditions for growing *H. Pylori* in *vitro* is necessary. However, on the other hand, since the results of many other kinds of molecular biological tests favored the correlation between these *Helicobacter* species and biliary lithiasis, whether only alive bacteria would cause stone disease or the biological component of *Helicobacter* itself is immunologically enough to be the initiator of evil for biliary lithiasis is also worth further investigation. In addition, our findings also suggested that performing multiple detection tests could increase the detection rate of *H. Pylori*. Although PCR is the most widely used technology for confirming the presence of *H. Pylori* due to its highly sensitivity, the detection rate was 25.41% in lithiasis group. Subgroup analysis of these studies showed that using only one test might not have enough power to confirm the relationship between the infection of *H. Pylori* and the biliary lithiasis (P = 0.26). In contrast, when performing at least two tests, the detection rate increased to 33.22% in lithiasis group which was significantly higher than that in control group (33.22% versus 16.20%, P = 0.004).

The prevalence of *Helicobacter pylori* infection ranges widely between nation and nation. In developing countries such as India, Pakistan, Latin America and Africna, the infection rate is approximately 80% of the population by 20 years of age. In contrast, this rate is as low as 10–20% in developed countries [50], [51]. The lower prevalence of *H. Pylori* in industrialized countries is attributed to the higher hygiene and socioeconomic standards [51]. In our meta-analysis, data showed a trend of higher presence of *H. Pylori* in cholelithiasis patients than control group and this trend was significant in the developing regions with higher prevalence of this bacterium. Notably, Japan is a special case. According to our study, although Japan is a developed country which has a higher standard of environmental hygiene, studies from this countries all reported relatively higher infection rates of *Helicobacter* from 30% to 60% in lithiasis patients and each indicated a strong correlation between the bacteria infection and biliary lithiasis [26], [31], [32], [36]. Based on browsing more literatures, we found the possible explanations. Interestingly, the younger generation in Japan have a similar infection rate as seen in developed countries whereas the older generation has the higher prevalence as seen in developing countries. For example, the infection rate of *H. pylori* in children under 10 years old is less than 5% and it gradually increased to approximately 20% in people of 10–19 years old, 25% in those 20–29 years old, 40% in those 30–39 years old and this rate is more than 60% at people of 50 years old and over [52]. Why this occurs might be due to the fact that higher standard of sanitary conditions such as clean public water systems were introduced in Japan after the 1950s. In our meta-analysis, the mean age of the biliary lithiasis patients of the studies from Japan was 50–60 years old, which is in consistent with the corresponding infection rate of the bacteria.

Besides *H. Pylori*, several other *Helicobacter* sp. including *H. Hepaticus*, *H. bilis H. Pullorum* and *H. Ganmani* have also been shown to be bile-tolerant and colonize the hepatobiliary system [9], [26], [31], [32], [35], [36]. Maurer et al reported that mice infected with *H. bilis* or co-infected with *H. Hepaticus* developed cholesterol gallstones at 80% prevalence compared with approximately 10% in uninfected controls [53]. *H. Ganmani* and *H. Pullorum* were novel enterohepatic *Helicobacter* species found in patients with cholestasis and other chronic liver diseases [54], [55]. In this meta-analysis, studies on these four species all reported relatively higher positive rates in lithiasis group than control group and this rate of *H. Hepaticus* reached the significant level (P = 0.02). Considering these studies were limited by small sample sizes, it's no doubt that further research should be undertaken to determine their association with cholelithiasis.

Regarding the limitations of this study, there was obvious heterogeneity across the studies, which suggested a diversity of study design may cause a disturbing impact on the results. Firstly, there is a lack of gold standard for the selection of the controls. Most of the studies involved patients with severe obesity or liver transplantation donors as controls and from whom the gallbladder and bile were obtained during routine cholecystectomy. However, a couple of studies collected biliary samples from gastric cancer patients. Considering the infection of *H. Pylori* is strongly linked to the pathogenesis of gastric cancer and the ascending duodenum pathway might be the possible entry route of the bacteria into the hepatobiliary tree, whether the unknown infectious status of *H. Pylori* in biliary tract of gastric cancer patients might affect the comparison between the case and control groups needs further observation. Secondly, for identifying the existence of *Helicobacter*, some studies used only one detecting method while others used several. This might result in reporting different cumulative positive rates between studies and induce significant heterogeneity. Finally, few studies mentioned whether they used antibiotics prior to sample collection which might further inhibit the growth of the organisms and produce a false negative result.

In conclusion, this meta-analysis revealed a trend of higher presence of *H. Pylori* in cholelithiasis patients than control group and this trend was significant in the regions with higher prevalence of this infectious agent. Evidences supporting the association between the *H. Pylori* infection and biliary lithiasis could be found in either bile, biliary tissue or stone samples. Because sensitivity and specifity differ greatly among various detecting techniques, the gold standard for the identification of *Helicobacter* species in biliary system has yet to be established. Besides *H. Pylori*, there have been a couple of studies showing higher percentage of other *Helicobacter* species in cholelithiasis patients, however, due to the limited number of studies and their small sample sizes, further research should be conducted to investigate their possible relationship. Considering the obvious heterogeneity in our study, we encourage carrying out large-scale and multicenter studies for clarifying the association of *Helicobacter* species and biliary lithiasis diseases to conclusion.

## Supporting Information

Figure S1 **Flow diagram of study selection.**
(DOC)

Table S1 **PRISMA Checklist.**
(DOC)
