# Supplementary material for: Are Helicobacter Pylori and Other Helicobacter Species Infection Associated with Human Biliary Lithiasis? A Meta-Analysis
Source: PLoS One. 2011 Nov 8;6(11):e27390. doi: 10.1371/journal.pone.0027390 (PMC3210793; doi:10.1371/journal.pone.0027390)
Supplement: Figure S1 — Flow diagram of study selection. (DOC) [file pone.0027390.s001.doc]

   Flow of Included Studies
